# 奥妥珠单抗诱导的急性血小板减少症1例

**DOI:** 10.3760/cma.j.cn121090-20240823-00317

**Published:** 2024-11

**Authors:** 孔扬 李, 雪琳 窦, 瑾 路

**Affiliations:** 1 北京大学人民医院血液科，北京大学血液病研究所，国家血液系统疾病临床医学研究中心，北京 100044 Peking University People's Hospital & Peking University Institute of Hematology, National Clinical Research Center for Hematologic Disease, Beijing 100044, China; 2 阳江市人民医院血液科，广东阳江 529500 Department of Hematology, People's Hospital of Yangjiang, Yangjiang 529500, China

患者，女，38岁。因“发现淋巴结肿大2年余”于2024年7月入院。患者2021年10月出现颈部多发肿物，1年后因腋窝及腹股沟出现多发肿物而就医。2022年11月PET-CT检查示“颈部双侧各区、双侧锁骨上/下区、前中纵隔、左内乳淋巴链、前肋角、双侧腋下、肝门区、肝胃间隙、胰周、小肠系膜、腹主动脉及双髂血管旁、双侧腹股沟多发代谢增高的淋巴结，大者位于肝门区，3.2 cm×2.9 cm，SUVmax 6.7；脾大且代谢增高，SUVmax 4.1”。随后行右侧腹股沟淋巴结粗针穿刺，病理示：淋巴组织内见多量淋巴细胞，免疫组织化学结果：CD21（显示FDC网），CD20（部分+），CD3（部分+），Ki67（大多数滤泡指数约20％，少量滤泡指数约30％），CD10（生发中心+），Bcl-6（生发中心+），BCL-2（生发中心−），CD30（散在+），MUM1（部分+），PD1（散在+），CXCL13（散在+），CD56（散在个别细胞+），颗粒酶B（−）。原位杂交结果：EBER（−）。结合免疫组织化学结果诊断为非霍奇金滤泡性淋巴瘤（Ⅰ～Ⅱ级），少量肿瘤性滤泡生长活跃。当时考虑无治疗指征，外院建议定期随诊观察。2024年6月患者出现胸闷至我院就诊，增强CT示：颈部、锁骨上、纵隔内、双侧腋下及右侧心膈角区多发肿大淋巴结（颈部较大者约1.4 cm×1.0 cm），右侧胸腔积液并右侧下叶压迫性肺不张，腹盆腔及腹膜后多发肿大淋巴结（大者约3.3 cm×2.6 cm）。患者无发热、盗汗、体重下降等不适。入院查体：颈部双侧可触及多个肿大淋巴结，较大者约1 cm×1 cm，质韧，无触痛，活动度尚可。右肺呼吸音减弱，未闻及干湿啰音。心律齐，未闻及杂音。腹软，无压痛及反跳痛，肝脾肋缘下未触及，双下肢无肿胀。入院查血常规：WBC 6.22×10^9^/L，HGB 146 g/L，PLT 130×10^9^/L。生化：肝细胞酶、总胆红素（TBIL）、乳酸脱氢酶（LDH）、血肌酐、总蛋白（TP）、白蛋白（ALB）等基本正常。β_2_微球蛋白2.8 mg/L。骨髓象：增生Ⅲ级，粒系占40％，红系占28.5％，粒红比1.4∶1；淋巴细胞占22％，其中约6％的淋巴细胞胞体小、胞质量少、核有切迹；全片见巨核细胞41个，血小板散在可见，未见病态造血或噬血现象。骨髓流式细胞术：异常单克隆B淋巴细胞占有核细胞3.89％，表达CD19、CD10dim、CD20、mKappa、cBcl-2str和CXCR4，部分表达CD11c、CD23和CD33，不表达CD81、ROR1、CD43、CD103、CD200、mLambda、CD5、CD38、CD123、CD34、CD138、FMC7、CD25、CD7和cKi67，考虑B细胞非霍奇金淋巴瘤。骨髓病理：淋巴细胞散在，可见灶性增生；免疫组化：CD3（−）、CD5（−）、CD19（+）、CD20（+）、CD79a（+）、CD21（−）、CD23（部分+）、CD103（−）、Bcl-2（+）、Bcl-6（−）、CyclinD1（−）、SOX11（−）、LEF1（散在+）、Ki67（<5％）、CD10（部分+），结合免疫表型支持滤泡性淋巴瘤。临床诊断：滤泡性淋巴瘤（病理分级Ⅰ～Ⅱ级，Ann Arbor分期Ⅳ期A组，FLIPI-1评分2分中危组，FLIPI-2评分1分低危组）。因患者出现胸腔积液，于2024年7月开始予GB方案（奥妥珠单抗1000 mg第1、8、15天，苯达莫司汀70 mg/m^2^第2、3天）化疗。第1剂奥妥珠单抗治疗后第2天，患者血小板计数降至37×10^9^/L，第4天降至27×10^9^/L，当时无出血表现，给予临床观察。第1剂奥妥珠单抗治疗后第8天患者血小板计数恢复至102×10^9^/L，按计划予以第2剂奥妥珠单抗，输注后第1天再次出现血小板减少（PLT 15×10^9^/L），输注血小板治疗有效，第15天PLT 50×10^9^/L，第29天PLT 95×10^9^/L，期间无严重出血表现。第2疗程给予BR方案（利妥昔单抗+苯达莫司汀）化疗，未发生血小板减少。

讨论：奥妥珠单抗是一种新型的人源化Ⅱ型抗CD20单抗，相对于人鼠嵌合型的Ⅰ型抗CD20单抗，其直接杀伤B细胞作用更强。奥妥珠单抗诱导的急性血小板减少症（OIAT）是指奥妥珠单抗输注后数天内发生的血小板减少症，具体机制尚不明确，国内暂未见这种严重不良事件的报道。通过提高对OIAT的重视、加强对使用奥妥珠单抗治疗患者的血小板水平监测，可做到早期诊断OIAT，视血小板下降程度予以血小板输注支持及转换为利妥昔单抗等干预措施。

